# Benchmarking the pediatric quality of life (PedsQL) cancer module in a large Dutch national cohort of childhood cancer patients

**DOI:** 10.1186/s12885-025-14322-6

**Published:** 2025-05-21

**Authors:** Elin Irestorm, Anne Bakker, Wim J. E. Tissing, Heleen Maurice-Stam, Natasja Dors, Annelies Mavinkurve-Groothuis, Sabine L. A. Plasschaert, Kelly L. A. van Bindsbergen, Martha A. Grootenhuis, Raphaele R. L. van Litsenburg

**Affiliations:** 1 Faculty of Medicine, Department of Paediatrics, Lund University, Lund, Sweden; 2 Princess Máxima Center for pediatric oncology, Utrecht, The Netherlands; 3 Faculty of Medicine, Utrecht University, Utrecht, The Netherlands

**Keywords:** Childhood cancer, Cancer treatment, (Health-Related) Quality of Life, PedsQL Cancer Module, Benchmark or reference values, Symptom

## Abstract

**Background:**

Pediatric cancer diagnosis and treatment reduce Health-Related Quality of Life (HRQoL). The Pediatric Quality of Life (PedsQL) Cancer Module is the most commonly used cancer specific HRQoL instrument, but most studies included relatively small cohorts and no reference scores are available. The development of a PedsQL Cancer Module benchmark, based on a large population of pediatric cancer patients, may benefit future research by providing insight into issues children encounter during treatment or clinical trials. A benchmark would also allow for earlier identification of patients who may benefit from targeted interventions in HRQoL monitoring programs. The primary aim of this study was therefore to provide benchmark scores from a large national cohort for use in pediatric oncology care and research. Secondary aims were to compare scores between subgroups (age, sex, and diagnosis group) and to describe the prevalence of impaired HRQoL.

**Methods:**

Data was collected from a national HRQoL monitoring program and extracted from medical records. HRQoL was measured with the PedsQL Cancer Module during treatment for childhood cancer, approximately 3 months after diagnosis. Comparisons were made for sex, age and diagnosis groups: hemato-oncology (HO), central nervous system-tumors (CNS), and solid tumors (ST) using the Mann-Whitney U test for the different subscales. Effect sizes were calculated. Impairment was defined on item level using descriptive statistics.

**Results:**

Proxy-reports of 492 children (age 2–7 years) and self-reports of 500 children (age 8–18 years) were available. HRQoL differences between age groups, sex and diagnosis groups were small, with the exception of nausea. On the nausea subscale there was a medium effect size difference with children with ST reporting more problems than children with CNS. Impaired HRQoL was most often reported on items reflecting nausea and procedural anxiety.

**Conclusions:**

Early in the cancer trajectory, there are only minor differences between subgroups based on age, sex, and diagnosis group. These results from a large national cohort can be used as benchmark data in future clinical trials, studies and clinical assessments, and offer and adapt support targeted for improving HRQoL related to treatment for childhood cancer.

**Supplementary Information:**

The online version contains supplementary material available at 10.1186/s12885-025-14322-6.

## Background

Over the years, treatment for childhood cancer patients has improved and hence increased survival [1, 2]. Not only have conventional treatment approaches become more effective, but also novel approaches have been incorporated into treatment protocols [3]. Due to improved treatment as well as improved supportive care [1], the five year survival rate of pediatric patients with cancer has increased to approximately 80% [2, 4]. Moreover, in high-income countries survival rates of pediatric patients with acute lymphoblastic leukemia (ALL) have now reached 90% [2, 5].

Although mortality rates have decreased, a pediatric cancer diagnosis and treatment negatively impact several patient-reported outcomes such as anxiety, fatigue and Health-Related Quality of Life (HRQoL) [6, 7]. HRQoL is widely understood as a multidimensional concept including different domains which are related to the effect of health, illness and treatment on the outcome of quality of life [8–10]. According to the Psychosocial standards of care, psychosocial health should be monitored in pediatric cancer patients [11]. Previous research has shown that monitoring psychosocial health through Patient-Reported Outcome Measures (PROMs) increases HRQoL, communication and satisfaction with care [12–15].

There are multiple factors related to a reduced HRQoL during pediatric cancer treatment, but the results are inconsistent. For example, in a recent systematic review [7] it was shown that younger age of the child was associated with better as well as worse pain-related HRQoL. Similar results were found for female sex regarding physical HRQoL. Results regarding differences between diagnosis are also conflicting, and many studies have focused on specific diagnoses such as ALL [7]. The inconsistencies are possibly due to different classifications of subgroups and limited sample sizes. In addition, there is a variety of PROMs that are in use. This also hampers clear conclusions about factors related to HRQoL.

One of the most commonly used PROMs to measure disease-specific HRQoL during treatment is the Pediatric Quality of Life Inventory (PedsQL) Cancer Module [7]. This PROM has been translated into over 90 languages [16] and psychometric properties are generally good [17, 18]. This cancer specific PROM assesses domains relevant to children with cancer, which in comparison to a generic PROM does not provide reference scores. Hence, many studies up to now have been limited to the comparison of subgroups within study cohorts. For example, the PedsQL Cancer Module has been used to describe differences between parent proxy-reports and child self-reports [19], between children on and off treatment [17], to compare HRQoL scores between different countries [20], or to describe the effect of an intervention on HRQoL [21, 22]. The development of a PedsQL Cancer Module benchmark, based on a large population of pediatric cancer patients, may benefit future developments in the field. It could, for example, provide comparison data for clinical trials and other studies that evaluate new interventions. A benchmark would also allow for earlier identification of patients who may benefit from targeted interventions to improve HRQoL when the PedsQL Cancer Module is used as HRQoL monitoring tool [11].

This study therefore evaluated HRQoL as measured with the PedsQL Cancer Module in a large Dutch cohort of children with cancer. The primary aim was to provide benchmark scores for use in pediatric oncology care and research. Secondary aims were to compare scores between subgroups defined by age, sex, and diagnosis group (hemato-oncology (HO), central nervous system-tumors (CNS), and solid tumors (ST)), and to describe the prevalence of impaired HRQoL in the whole cohort.

## Methods

### Participants and materials

Participants in the study were patients above 2 years of age that were treated at the Princess Máxima Center for pediatric oncology in Utrecht, the national center for childhood cancer in the Netherlands. Dutch and English speaking families are offered to participate in a patient-reported outcome monitoring program on quality of life called the KLIK PROM program (www.hetklikt.nu), which is part of standard care. Monitoring with the PedsQL Cancer Module starts around 3 months after diagnosis and the assessment is combined with regular appointments at the outpatient clinic with a minimum interval of 3 months, for the duration of the child’s anti-cancer treatment. For children above the age of 8, self-reports are used and for younger children (ages 2–7), the parent-proxy reports are used. We previously found that approximately 70% of offered questionnaires were completed [23, 24]. Informed consent to use this data for research purposes is available in 90–95%.

Data were collected through the KLIK PROM program between August 2015 and June 2024. Only the first assessment using the PedsQL Cancer Module was used for this study, meaning that no patients with a relapse participated, with a maximum of 5 months after diagnosis. Information on demographic and disease characteristics was extracted from the electronic hospital records.

### Outcomes

The main outcome of this study was HRQoL during treatment for childhood cancer, which was measured with the PedsQL 3.0 Cancer Module [17]. This PROM consists of eight subscale scores: pain and hurt (2 items), nausea (5 items), procedural anxiety (3 items), treatment anxiety (3 items), worry (3 items), cognitive problems (3/4/5 items, depending on age version), perceived physical appearance (3 items), and communication (3 items). Items are scored using a 5-point Likert scale (“never a problem”, “almost never a problem”, “sometimes a problem”, “often a problem”, “almost always a problem”). Recall time is 1 week. Items are then rescored to a 0-100 scale, where higher scores indicate better HRQoL.

Data were extracted for all patients for whom informed consent was given. The use of data collected in care for research questions was approved by the ethics committee of Rotterdam (no. MEC-2016-739). Additionally, the scientific committee of the Princess Máxima Center for pediatric oncology approved the project.

### Statistical methods

All statistical analyses were conducted with SPSS version 29.

Demographic variables were given in frequencies, with means and standard deviations (SD) for age. Scores on the PedsQL Cancer Module subscales were calculated in means, SD, 95% confidence intervals, medians, and interquartile ranges (IQR, p25-p75). The parent proxy-reports and self-reports were analyzed separately. Several subgroups were defined. For age, the age categories were defined in accordance with the PedsQL Cancer Module (2–4 years, 5–7 years, 8–12 years, and 13–18 years). For diagnosis, patients were divided into three main diagnosis groups: HO, CNS, and ST.

To compare the scores of patients between the groups, the non-parametric Mann-Whitney U test was used due to the non-normal distribution of scores. To conduct post-hoc analyses on the significant differences between the three diagnosis groups, the Kruskal Wallis test was used. Effect sizes (*r*) were calculated for the pairwise tests with statistically significant differences. Effect sizes below 0.3 were considered small, between 0.3 and 0.5 medium, and above 0.5 large [25]. Only medium and large effect sizes were considered clinically relevant. Due to multiple testing in the comparison of diagnosis groups, a Bonferroni correction was applied for each outcome by multiplying significance values by three.

To assess the prevalence of impaired HRQoL during treatment, the results were reported on item level. If problems were reported “sometimes”, “often”, or “almost always”, HRQoL on that item was considered to be impaired [26].

## Results

### Participant demographics and description of PedsQL cancer module scores for benchmarking

In total, 492 parents and 500 children/adolescents were included in the study. Since only the first assessment was used, all participants were unique and no patient had both a proxy-report and a self-report. For both proxy-reports and self-reports, the HO groups were the largest (266 proxy-reports and 298 self-reports). For both type of reports, there were more male than female patients, but for the proxy-reports the majority of responders were female caregivers. Participant characteristics are described in Table 1.

**Table 1:** Participant characteristics, divided by type of respondent

	Proxy-reports (children 2–7 years)	Self-reports (children 8–18 years)
**Number of participants**	492	500
**Child sex, N (%)**		
Male	282 (57.3%)	300 (60.0%)
Female	210 (42.7%)	200 (40.0%)
**Proxy-reporter sex, N (%)**		
Female	380 (77.2%)	N/A
Male	112 (22.8%)	
**Mean child age at diagnosis, years (SD)**	4.31 (1.64)	13.41 (3.0)
**Time since diagnosis, years (SD)**	0.29 (0.11)	0.27 (0.12)
Diagnoses, N (%*)		
*Hemato-oncology*	*266 (54.1%)*	*298 (59.8%)*
Acute lymphoblastic leukemia	197 (40.0%)	102 (20.5%)
Acute myeloid leukemia	18 (3.7%)	28 (5.6%)
Hodgkin lymphoma	4 (0.8%)	103 (20.7%)
Langerhans cell histiocytosis	14 (2.8%)	5 (1.0%)
Leukemia other	1 (0.2%)	0 (0%)
Non-hodgkin lymphoma	32 (6.5%)	60 (12.0%)
*Solid tumors*	*161 (32.8%)*	*115 (23.0%)*
Bone tumor	8 (1.6%)	52 (10.4%)
Germ cell tumor non-central nervous system	5 (1.0%)	12 (2.4%)
Kidney tumor	62 (12.7%)	9 (1.8%)
Liver tumor	9 (1.8%)	5 (1.0%)
Neuroblastoma	36 (7.3%)	4 (0.8%)
Soft tissue tumor	39 (8.0%)	24 (4.8%)
Solid tumor other	2 (0.4%)	9 (1.8%)
*Central nervous system tumors*	*65 (13.2%)*	*85 (17.0%)*
CNS germ cell tumor	1 (0.2%)	9 (1.8%)
CNS other	0 (0.0%)	5 (1.0%)
Craniopharyngioma	0 (0.0%)	5 (1.0%)
Embryonal tumor	3 (0.6%)	1 (0.2%)
Ependymoma and choroid plexus papilloma	7 (1.4%)	5 (1.0%)
High grade gliomas	9 (1.8%)	17 (3.4%)
Low grade gliomas	24 (4.9%)	25 (5.0%)
Medulloblastoma	21 (4.3%)	18 (3.6%)

* numbers may not add up to 100% due to rounding of decimals

N/A not applicable; CNS central nervous system

Parent proxy-reported scores varied between 53.56 (procedural anxiety subscale) and 91.57 (worry subscale). Self-reported scores varied between 70.02 (nausea subscale) and 86.47 (treatment anxiety subscale). Parent proxy-report and self-report scores for the entire cohort are shown in Table 2.

### Comparisons between age groups

Table 2 describes scores per respondent group and the comparison between younger and older patients, within each respondent group. For the proxy-reports, older patients (ages 5–7) were reported to have higher scores, indicating a better HRQoL on the subscales pain and hurt (*p* =.027, *r* =.10), procedural anxiety (*p* = < 0.001, *r* =.15), and treatment anxiety (*p* =.005, *r* =.13), but lower scores on worry (*p* = < 0.001, *r*=-.22) than the younger patients (ages 2–4). All effect sizes were small. Procedural anxiety was the subscale with the lowest score for both younger and older patients. For the self-reports, older patients (ages 13–18) reported higher scores, indicating better HRQoL, for procedural anxiety (*p* <.001, *r* =.27), treatment anxiety (*p* <.001, *r* =.19), and communication (*p* <.001, *r* =.15), but lower scores on perceived physical appearance (*p* =.041, *r*=-.09), and worry (*p* <.001, *r*=-.16) than the younger patients (ages 8–12). All effect sizes were small. For patients aged 8–12, procedural anxiety was the subscale with the lowest mean scores, but for patients aged 13–18 years it was worry.

**Table 2:** Descriptive scores per type of respondent for the entire cohort and comparisons between age groups, together with effect sizes

	**Parent-proxy reports (all)**			**Parent-proxy reports per age group**							
**Subscale PedsQL** **Cancer module**	**Age 2–7 (** *N* ** = 492)**			**Age 2–4 (** *N* ** = 318)**			**Age 5–7 (** *N* ** = 174)**			**Difference between age groups**	
	***Mean*** ***(SD)***	***95% CI***	***Median*** ***[IQR]***	***Mean*** ***(SD)***	***95% CI***	***Median*** ***[IQR]***	***Mean*** ***(SD)***	***95% CI***	***Median*** ***[IQR]***	***Sig***	***Effect size r***
Pain and hurt	74.06 *(*21.73)	72.13-76.00	75.00 [62.50–87.50]	72.56 (21.75)	70.16–74.96	75.00 [50.00-87.50]	76.80 (21.49)	73.58–80.01	75.00 [62.50–100.00]	0.027	0.10
Nausea	68.68 (23.20)	66.62–70.73	70.00 [50.00–90.00]	67.42 (23.14)	64.88–69.97	70.00 [50.00–85.00]	70.98 (23.18)	67.51–74.45	75.00 [53.75-90.00]	ns	
Procedural anxiety	53.56 (34.27)	50.52–56.59	50.00 [25.00-83.33]	49.71 (34.09)	45.95–53.47	50.00 [16.67–83.33]	60.58 (33.56)	55.56–65.61	66.67 [33.33–93.75]	< 0.001	0.15
Treatment anxiety	77.61 (27.60)	75.16–80.05	91.67 [58.33–100.00]	75.00 (28.65)	71.84–78.16	83.33 [56.25–100.00]	82.38 (24.97)	78.64–86.11	100.00 [66.67–100.00]	0.005	0.13
Worry	91.57 (17.23)	90.04–93.09	100.00 [91.67–100.00]	93.11 (16.82)	91.25–94.96	100.00 [100.00-100.00]	88.75 (17.65)	86.11–91.39	100.00 [83.33–100.00]	< 0.001	− 0.22
Cognitive problems	81.11 (19.35)	79.40-82.82	83.33 [66.67–100.00]	82.00 (18.75)	79.93–84.07	83.33 [66.67–100.00]	79.49 (20.36)	76.44–82.54	87.50 [62.50–100.00]	ns	
Perceived physical appearance	87.97 (17.31)	86.44–89.51	100.00 [83.33–100.00]	88.76 (16.75)	86.91–90.61	100.00 [83.33–100.00]	86.54 (18.27)	83,81-89.28	91.67 [75.00-100.00]	ns	
Communication	72.61 (30.40)	69.92–75.30	83.33 [50.00-100.00]	71.51 (32.77)	67.90-75.13	83.33 [50.00-100.00]	74.62 (25.47)	70.81–78.43	75.00 [58.33–100.00]	ns	
	**Self-reports (all)**			**Self-reports per age group**							
**Subscale PedsQL** **Cancer module**	**Age 8–18 (** *N* ** = 500)**			**Age 8–12 (** *N* ** = 210)**			**Age 13–18 (** *N* ** = 290)**			**Difference between age groups**	
	*Mean* *(SD)*	*95% CI*	*Median* *[IQR]*	*Mean* *(SD)*	*95% CI*	*Median* *[IQR]*	*Mean* *(SD)*	*95% CI*	*Median* *[IQR]*	*Sig*	*Effect size r*
Pain and hurt	71.50 (22.89)	69.49-73-51	75.00 [50.00-87.50]	72.74 (22.93)	69.62–75.86	75.00 [62.50–100.00]	70.60 (22.85)	67.96–73.24	75.00 [50.00-87.50]	ns	
Nausea	70.02 (23.24)	67.98–72.06	70.00 [55.00–90.00]	68.36 (24.59)	65.01–71.70	70.00 [50.00–90.00]	71.22 (22.18)	68.66–73.79	75.00 [55.00–90.00]	ns	
Procedural anxiety	72.30 (19.85)	69.71–74.89	83.33 [50.00-100.00]	62.98 (31.07)	58.75–67.20	66.67 [33.33–91.67]	79.05 (26.29)	76.01–82.09	91.67 [66.67–100.00]	< 0.001	0.27
Treatment anxiety	86.47 (19.85)	84.72–88.21	100.00 [75.00-100.00]	81.75 (22.87)	78.63–84.86	91.67 [66.67–100.00]	89.89 (16.56)	87.97–91.80	100.00 [83.33–100.00]	< 0.001	0.19
Worry	72.25 (22.57)	70.27–74.23	75.00 [58.33–91.67]	76.19 (21.57)	73.26–79.12	83.33 [66.67–91.67]	69.40 (22.89)	66.75–72.04	66.67 [58.33–83.33]	< 0.001	− 0.16
Cognitive problems	71.05 (20.70)	69.23–72.87	75.00 [60.00–85.00]	71.02 (20.60)	68.22–73.83	75.00 [55.00-86.25]	71.07 (20.82)	68.66–73.47	75.00 [60.00–85.00]	ns	
Perceived physical appearance	77.03 (21.50)	75.14–78.92	83.33 [66.67–91.67]	79.48 (19.95)	76.77–82.20	83.33 [66.67–100.00]	75.26 (22.41)	72.67–77.85	83.33 [66.67–91.67]	0.041	− 0.09
Communication	77.67 (22.96)	75.65–79.68	83.33 [66.67–100.00]	73.02 (25.21)	69.59–76.45	75.00 [50.00-93.75]	81.03 (20.58)	78.66–83.41	83.33 [75.00-100.00]	< 0.001	0.15

Effect sizes, measured with *r*, below 0.3 are considered small. between 0.3 and 0.5 are medium. and above 0.5 are large

Ns = not significant, SD = standard deviation, CI = confidence interval, IQR = interquartile range p25-p75

### Comparisons between sex

Table 3 describes the comparison between male and female patients, within each respondent group. For the parent proxy-reports, the only difference was on the worry subscale (*p* =.049, *r* =.09), where female patients were reported to have significantly better HRQoL, but the effect size was small. For the self-reports, female patients reported significantly lower scores indicating lower HRQoL on the subscales pain and hurt (*p* =.029, *r*=-.10), procedural anxiety (*p* =.003, *r*=-.14), worry (*p* =.003, *r*=-.13), cognitive problems (*p* =.026, *r*=-.10), perceived physical appearance (*p* <.001, *r*=-.17), and communication (*p* =.048, *r*=-.09), but all effect sizes were small.

### Comparisons between diagnosis groups

Table 4 describes the comparison of the parent proxy-reported scores between patients in the HO-, ST- and CNS-groups. There were no significant differences between the diagnosis groups regarding treatment anxiety, worry, perceived physical appearance, or communication. For the subscales with significant differences, all effect sizes were small.

HO patients had significantly higher scores for procedural anxiety compared to patients with ST (*p* =.007, *r* =.14). The HO group did report significantly lower scores, indicating lower HRQoL, for pain and hurt (*p* =.017, *r* =.12) compared to patients with a CNS tumor. ST-patients had significantly lower scores on nausea than the CNS-group (*p* =.007, *r* =.14). Children diagnosed with a CNS-tumor had significantly lower scores for the cognitive problems subscale compared to both HO-patients (*p* <.001, *r* =.19) and ST-patients (*p* <.001, *r* =.24).

Table 5 describes the comparison of the self-reported scores between patients in the HO-, ST- and CNS-groups. There were no significant differences between the diagnosis groups on the subscales procedural anxiety, worry, cognitive problems, and communication. Patients in the HO-group scored lower on the subscales pain and hurt (*p* <.001, *r* =.23) and nausea (*p* <.001, *r* =.23) than the CNS-group, indicating lower HRQoL. They also scored lower on perceived physical appearance compared to both CNS-patients (*p* =.005, *r* =.16) and ST-patients (*p* =.037, *r* =.12). ST-patients had significantly lower scores on the nausea subscale than CNS-patients (*p* = < 0.001, *r* =.36). They also reported significantly lower scores for treatment anxiety (*p* =.005, *r* =.15) than HO-patients. The effect sizes were small for all subscales except for the nausea subscale, where there was a medium effect size for the difference between patients with ST compared to those with a CNS tumor.

**Table 3:** Descriptive scores for different subscales and comparisons between male and female patients (parent-proxy reports and self-reports), together with effect sizes

	**Parent-proxy reports (age 2–7)**							
**Subscale PedsQL Cancer module**	**Male (** *N* ** = 282)**			**Female (** *N* ** = 210)**			**Difference between** **males and females**	
	*Mean* *(SD)*	*95% CI*	*Median* *[IQR]*	*Mean* *(SD)*	*95% CI*	*Median* *[IQR]*	*Sig*	*Effect size r*
Pain and hurt	74.42 (21.33)	71.92–76.92	75.00 [62.50–87.50]	73.57 (22.30)	70.54–76.61	75.00 [50.00-100.00]	ns	
Nausea	67.62 (23.46)	64.87–70.37	70.00 [50.00–85.00]	70.10 (22.81)	66.99–73.20	75.00 [55.00–90.00]	ns	
Procedural anxiety	52.16 (34.23)	48.14–56.17	50.00 [25.00-83.33]	55.44 (34.30)	50.77–60.10	58.33 [25.00-83.33]	ns	
Treatment anxiety	76.60 (28.45)	73.26–79.93	91.67 [58.33–100.00]	78.97 (26.43)	75.37–82.56	91.67 [64.59–100.00]	ns	
Worry	90.54 (18.61)	88.36–92.72	100.00 [91.67–100.00]	92.94 (15.11)	90.88–94.99	100.00 [91.67–100.00]	0.049	0.09
Cognitive problems	80.45 (20.34)	78.07–82.84	85.42 [66.67–100.00]	81.99 (17.94)	79.55–84.43	83.33 [66.67–100.00]	ns	
Perceived physical appearance	88.48 (16.95)	86.49–90.46	100.00 [83.33–100.00]	87.30 (17.81)	84.88–89.72	100.00 [75.00-100.00]	ns	
Communication	71.07 (31.30)	67.40-74.74	83.33 [50.00-100.00]	74.68 (29.08)	70.73–78.64	83.33 [58.33–100.00]	ns	
	**Self-reports (age 8–18)**							
**Subscale PedsQL** **Cancer module**	**Male (** *N* ** = 300)**			**Female (** *N* ** = 200)**			**Difference between** **males and females**	
	***Mean*** ***(SD)***	***95% CI***	***Median*** ***[IQR]***	***Mean*** ***(SD)***	***95% CI***	***Median*** ***[IQR]***	***Sig***	***Effect size r***
Pain and hurt	73.42 (21.99)	70.92–75.91	75.00 [62.50–87.50]	68.62 (23.94)	65.29–71.96	62.50 [50.00-87.50]	0.029	− 0.10
Nausea	70.20 (23.33)	67.55–72.85	75.00 [55.00–90.00]	69.75 (23.16)	66.52–72.98	70.00 [55.00–90.00]	ns	
Procedural anxiety	75.39 (27.92)	72.22–78.56	83.33 [58.33–100.00]	67.67 (31.12)	63.33–72.01	75.00 [50.00-91.67]	0.003	− 0.13
Treatment anxiety	87.58 (19.52)	85.37–89.80	100.00 [83.33–100.00]	84.79 (20.27)	81.97–87.62	91.67 [75.00-100.00]	ns	
Worry	74.64 (21.57)	72.19–77.09	75.00 [66.67–91.67]	68.67 (23.60)	65.38–71.96	66.67 [50.00-83.33]	0.003	− 0.13
Cognitive problems	72.83 (19.64)	70.60-75.06	75.00 [60.00-88.75]	68.38 (21.98)	65.31–71.44	70.00 [55.00–85.00]	0.026	− 0.10
Perceived physical appearance	79.89 (20.20)	77.59–82.18	83.33 [66.67–100.00]	72.75 (22.69)	69.59–75.91	75.00 [58.33–91.67]	< 0.001	− 0.17
Communication	79.19 (22.59)	76.63–81.76	83.33 [66.67–83.33]	75.38 (23.37)	72.12–78.64	83.33 [58.33–91.67]	0.048	− 0.09

Effect sizes, measured with *r*, below 0.3 are considered small. between 0.3 and 0.5 are medium. and above 0.5 are large. Ns = not significant, SD = standard deviation, CI = confidence interval, IQR = interquartile range p25-p75

**Table 4:** Descriptive scores for different subscales and comparisons between diagnostic groups for parent-proxy reports, together with effect sizes

Parent-proxy reports (age 2–7)												
Subscale PedsQL Cancer module	Hemato-oncology (*N* = 266)			Solid tumors (*N* = 161)			Central nervous system tumors (*N* = 65)			Comparisons between groups		
	*Mean* *(SD)*	*95% CI*	*Median* *[IQR]*	*Mean* *(SD)*	*95% CI*	*Median* *[IQR]*	*Mean* *(SD)*	*95% CI*	*Median* *[IQR]*	*post hoc analysis*	Sig*	*Effect size r*
Pain and hurt	72.13 (23.25)	69.33–74.94	75.00 [50.00-87.50]	74.46 (19.78)	71.38–77.54	75.00 [62.50–87.50]	80.96 (18.50)	76.38–85.55	87.50 [75.00-100.00]	HO vs. ST HO vs. CNS ST vs. CNS	0.100 0.017 0.089	− 0.12
Nausea	68.99	66.19–71.78	70.00	65.84	62.40-69.27	70.00	74.46	68.23–80.70	85.00	HO vs. ST	0.353	
	(23.17)		[53.75-90.00]	(22.07)		[50.00–85.00]	(25.16)		[55.00–95.00]	HO vs. CNS	0.110	
										ST vs. CNS	0.007	− 0.14
Procedural anxiety	57.39 (33.34)	53.37–61.42	58.33 [33.33–91.67]	47.15 (34.46)	41.79–52.52	50.00 [16.67-75.00]	53.72 (35.69)	44.87–62.56	50.00 [25.00-87.50]	HO vs. ST HO vs. CNS ST vs. CNS	0.007 1.000 0.524	0.14
Treatment anxiety	80.42 (26.29)	77.25–83.59	100.00 [66.67–100.00]	75.05 (28.37)	70.64–79.47	83.33 [58.33–100.00]	72.44 (29.90)	65.03–79.84	83.33 [50.00-100.00]	HO vs. ST HO vs. CNS ST vs. CNS	0.086 0.071 1.000	
Worry	92.89 (15.44)	91.03–94.75	100.00 [91.67–100.00]	89.75 (19.68)	86.69–92.81	100.00 [83.33–100.00]	90.64 (17.46)	86.31–94.97	100.00 [83.33–100.00]	ns		
Cognitive problems	81.39	79.11–83.67	83.33	85.18	82.39–87.97	91.67	69.87	64.76–74.99	75.00	HO vs. ST	0.098	
	(18.86)		[66.67–100.00]	(17.93)		[75.00-100.00]	(20.64)		[56.25–83.33]	HO vs. CNS	< 0.001	0.19
										ST vs. CNS	< 0.001	0.24
Perceived physical appearance	88.22 (17.42)	86.12–90.32	100.00 [83.33–100.00]	87.73 (17.26)	85.05–90.42	100.00 [75.00-100.00]	87.56 (17.25)	83.29–91.84	100.00 [75.00-100.00]	ns		
Communication	74.12 (28.75)	70.65–77.59	83.33 [58.33–100.00]	71.38 (32.74)	66.28–76.47	83.33 [50.00-100.00]	69.49 (31.02)	61.8-77.17	75.00 [45.84–100.00]	ns		

Comparisons between the 3 groups were made with the non-parametric Kruskal-Wallis test. When a significant difference was detected. a post-hoc analysis between the different groups was conducted with Mann-Whitney U. *Significance values have been adjusted by the Bonferroni correction for multiple testing, multiplying the significance values by three. Effect sizes. measured with *r*, below 0.3 are considered small, between 0.3 and 0.5 are medium, and above 0.5 are large. Abbreviations: HO = Hemato-oncology; ST = Solid Tumors; CNS = Central Nervous System-tumors. Ns = not significant, SD = standard deviation, CI = confidence interval, IQR = interquartile range p25-p75

**Table 5:** Descriptive scores for different subscales and comparisons between diagnostic groups for child self-reports, together with effect sizes

Self-reports (age 8–18)												
Subscale PedsQL Cancer module	Hemato-oncology (*N* = 298)			Solid tumors (*N* = 115)			Central nervous system tumors (*N* = 85)			Comparisons between groups		
	*Mean* *(SD)*	95% CI	Median [IQR]	*Mean* *(SD)*	95% CI	Median [IQR]	*Mean* *(SD)*	95% CI	Median [IQR[	*post hoc analysis*	Sig*	*Effect size r*
Pain and hurt	68.67 (23.28)	66.01–71.32	68.75 [50.00-87.50]	73.7 (22.11)	69.61–77.78	75.00 [62.50–87.50]	79.12 (20.27)	74.75–83.49	87.50 [62.50–100.00]	HO vs. ST HO vs. CNS ST vs. CNS	0.113 < 0.001 0.270	− 0.23
Nausea	69.46 (22.41)	66.91–72.02	70.00 [55.00–90.00]	63.78 (24.40)	59.27–68.29	65.00 [45.00–85.00]	79.94 (21.47)	75.31–84.57	90.00 [65.00-100.00]	HO vs. ST HO vs. CNS ST vs. CNS	0.115 < 0.001 < 0.001	− 0.23 − 0.36
Procedural anxiety	73.97 (28.28)	70.73–77.20	83.33 [50.00-100.00]	70.51 (30.69)	64.84–76.18	83.33 [41.67–100.00]	69.61 (31.28)	62.86–76.35	83.33 [45.84–100.00]	ns		
Treatment anxiety	88.39 (18.81)	86.25–90.54	100.00 [83.33–100.00]	82.10 (21.80)	78.07–86.13	91.67 [66.67–100.00]	85.69 (19.95)	81.38–89.99	100.00 [75.00-100.00]	HO vs. ST HO vs. CNS ST vs. CNS	0.005 0.748 0.450	0.15
Worry	71.84 (22.21)	69.31–74.37	75.00 [58.33–91.67]	70.22 (24.01)	65.78–74.65	75.00 [50.00-91.67]	76.47 (21.33)	71.87–81.07	83.33 [66.67–95.84]	ns		
Cognitive problems	71.80 (19.10)	69.62–73.97	72.50 [60.00–85.00]	70.74 (22.58)	66.57–74.91	75.00 [60.00–85.00]	69.88 (22.68)	64.99–74.77	75.00 [55.00–90.00]	ns		
Perceived physical appearance	74.22 (22.57)	71.64–76.79	75.00 [58.33–91.67]	80.00 (20.35)	76.24–83.76	83.33 [66.67–100.00]	82.84 (17.22)	79.13–86.56	83.33 [75.00-100.00]	HO vs. ST HO vs. CNS ST vs. CNS	0.037 0.005 1.00	− 0.12 − 0.16
Communication	78.08 (22.77)	75.48–80.67	83.33 [66.67–100.00]	77.25 (22.25)	73.14–81.36	83.33 [66.67–100.00]	76.67 (24.93)	71.29–82.04	83.33 [66.67–100.00]	ns		

Comparisons between the 3 groups were made with the non-parametric Kruskal-Wallis test. When a significant difference was detected. a post-hoc analysis between the different groups was conducted with Mann-Whitney U. * Significance values have been adjusted by the Bonferroni correction for multiple testing multiplying the significance values by three. Effect sizes, measured with *r*, below 0.3 are considered small. between 0.3 and 0.5 are medium. and above 0.5 are large. Abbreviations: HO = Hemato-oncology; ST = Solid Tumors; CNS = Central Nervous System-tumors. Ns = not significant, SD = standard deviation, CI = confidence interval, IQR = interquartile range p25-p75

### Impairments in HRQoL

**Fig. 1:**
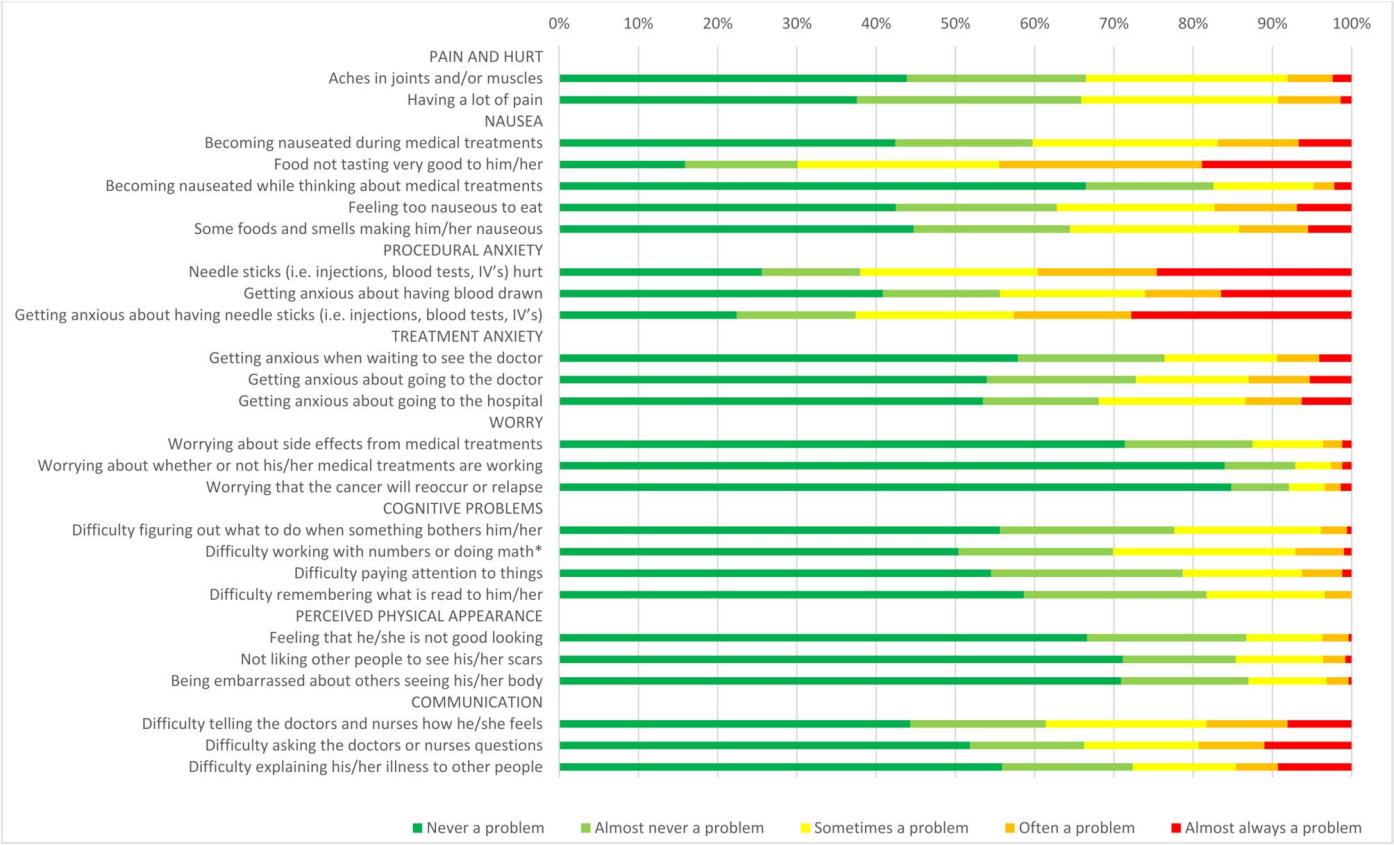
Proxy-reported cancer-specific problems (age 2–7 years), *N* = 492. *Only administered to ages 5–7 years

**Fig. 2:**
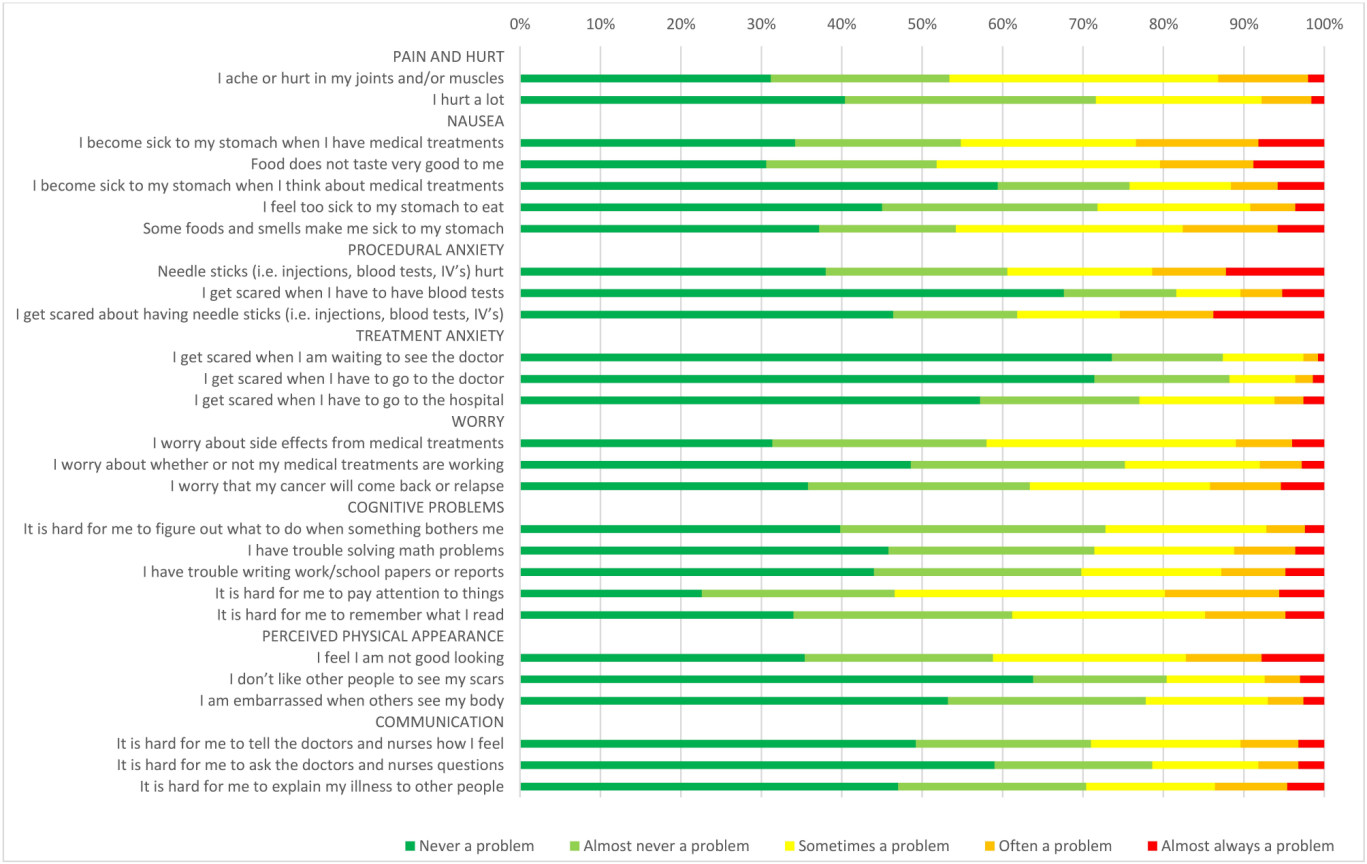
Self-reported cancer-specific problems (age 8–18 years), *N* = 500

Results on item level are reported in Fig. 1 for parent-reports and Fig. 2 for self-reports, and in supplemental Tables 1 and 2.

According to parent-reports, the 3 items where impairment most often occurred were part of the nausea and the procedural anxiety subscales: “Food not tasting very good to him/her” (70%), “Getting anxious about having needle sticks” (63%), and “Needle sticks hurt” (62%). Items that were most often indicated to never be a problem were all part of the worry subscale: “Worrying that the cancer will reoccur or relapse” (85%), “Worrying about whether or not his/her medical treatments are working” (84%), and “Worrying about side effects from medical treatments” (71%).

According to self-reports, the 3 items where impairment most often occurred were part of the cognitive problems, nausea, and pain and hurt subscales: “It is hard for me to pay attention to things” (53%), “Food does not taste very good to me” (48%), and “I ache or hurt in my joints and/or muscles” (47%). Items that were most often indicated to never be a problem were part of the treatment anxiety and the procedural anxiety subscales: “I get scared when I am waiting to see the doctor” (74%), “I get scared when I have to go to the doctor” (71%), and “I get scared when I have to have blood tests” (68%).

## Discussion

In this study, we described scores on the PedsQL Cancer Module from a large Dutch national cohort undergoing treatment for childhood cancer between August 2015 and June 2024. This benchmark can serve as a reference for future clinical trials, studies and clinical assessments. When using this data as reference, either the subscale scores or the responses on item level can be used.

We compared subgroups based on age, sex, and main diagnosis group. Regarding age, we reported several statistically significant differences between younger and older children, but with small effect sizes. Considering the effect sizes, the results indicate that age is not an important concern when choosing reference values. Relatedly, while we reported several statistical differences between males and females, especially for the self-reports, all the effect sizes were small. This implies that generally, no distinction has to be made for sex when choosing reference values.

Although significant differences were found between the three diagnosis groups, all but one of the effect sizes were small, indicating that these differences are also not very clinically relevant early in the treatment trajectory. The only medium effect size was found for the nausea subscale, where self-reported scores indicated more problems in patients with ST compared to patients with a CNS-tumor. More nausea in patients with ST have also previously been reported during treatment, likely related to more emetogenic chemotherapy. These problems were more serious 6 months after diagnosis than 3 months after diagnosis [27]. The general lack of clinically relevant differences fits the expectation that at this early stage of treatment, when all participants are receiving anti-cancer treatment, there are no large differences between diagnosis groups on the subscales assessed by the PedsQL Cancer Module.

The lack of clinically relevant differences between ages, sex and diagnosis groups implies that the overall references scores as presented in Table 2 can be used as reference values for studies with mixed diagnosis populations, as the included cohort is representative for the expected distribution of childhood cancer diagnoses [28]. However, some aspects need to be considered. Our study reflects HRQoL during the early stages of treatment. The differences between subgroups may change over time. For example, previous research has shown that the HRQoL of patients treated for ALL significantly improves during treatment on certain subscales, such as nausea and communication [29]. The effect of specific treatments or adverse effects was also not captured in this study. Children with ALL for example report significantly reduced scores on the Cancer Module during treatment with dexamethasone compared to without dexamethasone [30, 31]. As cyclic treatment with glucocorticoids is not scheduled in the first five months in Dutch ALL protocols, the effect of this treatment is not reflected in the current study. Additionally, our results do not necessarily imply that differences are absent altogether. For example, in patients with a CNS tumor cognitive deficits have previously been reported in neuropsychologic assessments already before the start of treatment [32]. However, in this study, this did not translate to differences with a medium or large effect size between diagnosis groups on the cognitive problems subscale. This might be related to the study sample’s characteristics, with a relatively large proportion of lower grade lesions. Last but not least, we reported on data collected in a hospital with many resources available for children and parents both in terms of the care model (a focus on preventing medical traumatic stress) materials, as well as in terms of HRQoL services (supportive care, psycho-oncology care) which might have an influence on the reported HRQoL [31].

Regarding the prevalence of impaired HRQoL reported during treatment, we described prevalences for each item. Items from the procedural anxiety and nausea subscales were reported frequently as impaired. Procedural anxiety and nausea are therefore two very important areas for health care staff to consider during treatment. According to the psychosocial standards of care in pediatric oncology, all children should receive developmentally appropriate preparatory information about invasive medical procedures as well as psychological interventions for these procedures [33]. The psychosocial team at the Princess Máxima Center for pediatric oncology has dedicated child life specialists whenever these procedures need to be done, with an individualized plan to decrease anxiety and prevent medical traumatic stress. The low frequency of participants reporting problems with worry and treatment anxiety might be an indicator of a successful implementation of the care system. Despite this, procedural anxiety remains a problem in young children.

### Strengths and limitations

When possible, self-reports have advantages of proxy-reports within the field of childhood cancer [34]. While parent proxy-reports cannot replace self-reports, including those reports for age groups who cannot self-report is a strength of this study. Additionally, this study utilized data from the Dutch national cohort of children and adolescents with cancer, including almost 1,000 participants. However, some limitations must be mentioned as well. The assessments were conducted on average 3 months after the diagnosis, meaning that those with a shorter treatment period (mainly patients treated with either only surgery or only neurosurgery) were not participating. The second limitation is that only Dutch or English speaking families are invited to participate in the patient-reported outcome program. Furthermore, it is important to realize we only studied age, sex and diagnosis groups. Other risk or protective factors are important to include in future studies. From other studies we know parental distress is related to lower HRQoL [35, 36], but other factors such as new medical treatments should be considered as well to get more insight into disease specific HRQoL in pediatric oncology [7].

Finally, we did not report scores for a total scale score, even though in other studies this is frequently the only reported measure. The PedsQL Cancer Module was not developed to contain an overall score of all subscales. HRQoL is widely understood as a multidimensional concept including different domains [8, 9], which is stressed in this work where we show that some subscales are more impaired than others.

## Conclusions

The results from this study provide insight into HRQoL scores on the PedsQL Cancer Module in a large cohort of pediatric oncology patients within 5 months after diagnosis. Additionally, it provides information on the frequency of different problems experienced by children and adolescents undergoing treatment for cancer in the Netherlands. The results can be used as reference data for clinical trials, studies and clinical assessments in care. They can also be used to offer and adapt support targeted at improving HRQoL related to treatment for childhood cancer. Our results show that early in the cancer trajectory, there are only minor differences between subgroups based on diagnosis, age, and sex. Instead, specific subscales of HRQoL such as nausea and procedural anxiety are impaired, and focus should be on providing support in these areas.

## Electronic supplementary material

Below is the link to the electronic supplementary material.

Supplementary Material 1

## Data Availability

The data and code necessary to reproduce the analyses presented here will be made available, as will the materials necessary to attempt to replicate the findings. It will be made available after publication upon request to researchers who provide a methodologically sound proposal and after a signed data sharing agreement is reached. Proposals should be submitted to prom@prinsesmaximacentrum.nl.
