# Supplementary material for: Benchmarking the pediatric quality of life (PedsQL) cancer module in a large Dutch national cohort of childhood cancer patients
Source: BMC Cancer. 2025 May 21;25:915. doi: 10.1186/s12885-025-14322-6 (PMC12096553; doi:10.1186/s12885-025-14322-6)
Supplement: Supplementary file 1 — Supplementary Material 1 [file 12885_2025_14322_MOESM1_ESM.docx]

| **Supplemental table 1** Proxy-reported prevalence of cancer-specific problems (age 2-7 years), N=492. *****Only administered to ages 5-7 years. | | | | | |
| --- | --- | --- | --- | --- | --- |
|  | **Never a problem** | **Almost never a problem** | **Sometimes a problem** | **Often a problem** | **Almost always a problem** |
| **PAIN AND HURT** |  |  |  |  |  |
| Aches in joints and/or muscles | 43.9% | 22.6% | 25.4% | 5.7% | 2.4% |
| Having a lot of pain | 37.6% | 28.3% | 24.8% | 7.9% | 1.4% |
| **NAUSEA** |  |  |  |  |  |
| Becoming nauseated during medical treatments | 42.5% | 17.3% | 23.4% | 10.2% | 6.7% |
| Food not tasting very good to him/her | 15.9% | 14.2% | 25.4% | 25.6% | 18.9% |
| Becoming nauseated while thinking about medical treatments | 66.5% | 16.1% | 12.6% | 2.6% | 2.2% |
| Feeling too nauseous to eat | 42.5% | 20.3% | 19.9% | 10.4% | 6.9% |
| Some foods and smells making him/her nauseous | 44.7% | 19.7% | 21.3% | 8.7% | 5.5% |
| **PROCEDURAL ANXIETY** |  |  |  |  |  |
| Needle sticks (i.e. injections, blood tests, IV’s) hurt | 25.6% | 12.4% | 22.4% | 15.0% | 24.6% |
| Getting anxious about having blood drawn | 40.9% | 14.8% | 18.3% | 9.6% | 16.5% |
| Getting anxious about having needle sticks (i.e. injections, blood tests, IV’s) | 22.4% | 15.0% | 19.9% | 14.8% | 27.8% |
| **TREATMENT ANXIETY** |  |  |  |  |  |
| Getting anxious when waiting to see the doctor | 57.9% | 18.5% | 14.2% | 5.3% | 4.1% |
| Getting anxious about going to the doctor | 54.0% | 18.8% | 14.2% | 7.7% | 5.3% |
| Getting anxious about going to the hospital | 53.5% | 14.6% | 18.5% | 7.1% | 6.3% |
| **WORRY** |  |  |  |  |  |
| Worrying about side effects from medical treatments | 71.3% | 16.1% | 8.9% | 2.4% | 1.2% |
| Worrying about whether or not his/her medical treatments are working | 83.9% | 8.9% | 4.5% | 1.4% | 1.2% |
| Worrying that the cancer will reoccur or relapse | 84.8% | 7.3% | 4.5% | 2.0% | 1.4% |
| **COGNITIVE PROBLEMS** |  |  |  |  |  |
| Difficulty figuring out what to do when something bothers him/her | 55.7% | 22.0% | 18.5% | 3.3% | 0.6% |
| Difficulty working with numbers or doing math* | 50.4% | 19.5% | 23.0% | 6.1% | 1.0% |
| Difficulty paying attention to things | 54.5% | 24.2% | 15.0% | 5.1% | 1.2% |
| Difficulty remembering what is read to him/her | 58.6% | 23.0% | 14.9% | 3.4% | 0.0% |
| **PERCEIVED PHYSICAL APPEARANCE** |  |  |  |  |  |
| Feeling that he/she is not good looking | 66.7% | 20.1% | 9.6% | 3.3% | 0.4% |
| Not liking other people to see his/her scars | 71.1% | 14.2% | 11.0% | 2.8% | 0.8% |
| Being embarrassed about others seeing his/her body | 70.9% | 16.1% | 9.8% | 2.8% | 0.4% |
| **COMMUNICATION** |  |  |  |  |  |
| Difficulty telling the doctors and nurses how he/she feels | 44.3% | 17.1% | 20.3% | 10.2% | 8.1% |
| Difficulty asking the doctors or nurses questions | 51.8% | 14.4% | 14.4% | 8.3% | 11.0% |
| Difficulty explaining his/her illness to other people | 55.9% | 16.5% | 13.0% | 5.3% | 9.3% |

| **Supplemental table 2** Self-reported prevalence cancer-specific problems (age 8-18 years), N=500. | | | | | |
| --- | --- | --- | --- | --- | --- |
|  | **Never a problem** | **Almost never a problem** | **Sometimes a problem** | **Often a problem** | **Almost always a problem** |
| **PAIN AND HURT** |  |  |  |  |  |
| I ache or hurt in my joints and/or muscles | 31.2% | 22.2% | 33.4% | 11.2% | 2.0% |
| I hurt a lot | 40.4% | 31.2% | 20.6% | 6.2% | 1.6% |
| **NAUSEA** |  |  |  |  |  |
| I become sick to my stomach when I have medical treatments | 34.2% | 20.6% | 21.8% | 15.2% | 8.2% |
| Food does not taste very good to me | 30.6% | 21.2% | 27.8% | 11.6% | 8.8% |
| I become sick to my stomach when I think about medical treatments | 59.4% | 16.4% | 12.6% | 5.8% | 5.8% |
| I feel too sick to my stomach to eat | 45.0% | 26.8% | 19.0% | 5.6% | 3.6% |
| Some foods and smells make me sick to my stomach | 37.2% | 17.0% | 28.2% | 11.8% | 5.8% |
| **PROCEDURAL ANXIETY** |  |  |  |  |  |
| Needle sticks (i.e. injections, blood tests, IV’s) hurt | 38.0% | 22.6% | 18.0% | 9.2% | 12.2% |
| I get scared when I have to have blood tests | 67.6% | 14.0% | 8.0% | 5.2% | 5.2% |
| I get scared about having needle sticks (i.e. injections, blood tests, IV’s) | 46.4% | 15.4% | 12.8% | 11.6% | 13.8% |
| **TREATMENT ANXIETY** |  |  |  |  |  |
| I get scared when I am waiting to see the doctor | 73.6% | 13.8% | 10.0% | 1.8% | 0.8% |
| I get scared when I have to go to the doctor | 71.4% | 16.8% | 8.2% | 2.2% | 1.4% |
| I get scared when I have to go to the hospital | 57.2% | 19.8% | 16.8% | 3.6% | 2.6% |
| **WORRY** |  |  |  |  |  |
| I worry about side effects from medical treatments | 31.4% | 26.6% | 31.0% | 7.0% | 4.0% |
| I worry about whether or not my medical treatments are working | 48.6% | 26.6% | 16.8% | 5.2% | 2.8% |
| I worry that my cancer will come back or relapse | 35.8% | 27.6% | 22.4% | 8.8% | 5.4% |
| **COGNITIVE PROBLEMS** |  |  |  |  |  |
| It is hard for me to figure out what to do when something bothers me | 39.8% | 33.0% | 20.0% | 4.8% | 2.4% |
| I have trouble solving math problems | 45.8% | 25.6% | 17.4% | 7.6% | 3.6% |
| I have trouble writing work/school papers or reports | 44.0% | 25.8% | 17.4% | 8.0% | 4.8% |
| It is hard for me to pay attention to things | 22.6% | 24.0% | 33.6% | 14.2% | 5.6% |
| It is hard for me to remember what I read | 34.0% | 27.2% | 24.0% | 10.0% | 4.8% |
| **PERCEIVED PHYSICAL APPEARANCE** |  |  |  |  |  |
| I feel I am not good looking | 35.4% | 23.4% | 24.0% | 9.4% | 7.8% |
| I don’t like other people to see my scars | 63.8% | 16.6% | 12.2% | 4.4% | 3.0% |
| I am embarrassed when others see my body | 53.2% | 24.6% | 15.2% | 4.4% | 2.6% |
| **COMMUNICATION** |  |  |  |  |  |
| It is hard for me to tell the doctors and nurses how I feel | 49.2% | 21.8% | 18.6% | 7.2% | 3.2% |
| It is hard for me to ask the doctors and nurses questions | 59.0% | 19.6% | 13.2% | 5.0% | 3.2% |
| It is hard for me to explain my illness to other people | 47.0% | 23.4% | 16.0% | 9.0% | 4.6% |
